# Survival from lung cancer in England and Wales up to 2001

**DOI:** 10.1038/sj.bjc.6604584

**Published:** 2008-09-23

**Authors:** Y K Zee, T Eisen

**Affiliations:** 1 Department of Oncology, University of Cambridge, Box 193 Addenbrooke's Hospital, Hills Road, Cambridge CB2 0QQ, Cambridge, UK

With over 38 000 new cases each year, lung cancer is the second most common form of cancer in the United Kingdom after breast cancer (Cancer Research UK, 2008a) and it is the most common cancer cause of death (Cancer Research UK, 2008b). Tobacco smoking is undoubtedly the major aetiological risk factor, the risk being around ten times higher in long-term smokers compared with non-smokers (Doll and Peto, 1981). Men are more likely to be affected, although the number of women with lung cancer has been increasing. This reflects changes in smoking habits over the last century (Quinn *et al*, 2001). Smoking cessation before middle age avoids more than 90% of the lung cancer risk attributable to tobacco (Peto *et al*, 2000).

Lung cancer can be broadly classified into two main types: non-small-cell lung cancer (NSCLC), which accounts for about 80% of cases, and small-cell lung cancer (SCLC), which accounts for the other 20%. Approximately 70% of patients with NSCLC present with advanced (Stage III/IV) disease (Ihde and Minna, 1991; Ihde, 1992). At diagnosis, about 60% of patients with SCLC have extensive disease, defined as disease not contained within a hemithorax, with metastases involving one or more sites such as the brain, liver, bone or bone marrow (Carney, 2002).

## Standards of diagnosis between 1985 and 2000

No screening services are provided for early detection of cancer of the lung in England and Wales as there is currently no evidence that population screening is effective in reducing mortality (Manser *et al*, 2003). Most patients in the United Kingdom present to their primary care physician. Lung cancer should be suspected in any smoker with new or worsening respiratory symptoms including haemoptysis, or indication of systemic illness such as anorexia, malaise or weight loss (NICE, 2005). If a chest X-ray suggests lung cancer, then patients are offered urgent referral to a member of the lung cancer multidisciplinary team, usually a chest physician. The diagnosis may be confirmed from sputum cytology, bronchoscopy, percutaneous needle biopsy or open biopsy. Staging of lung cancers is by computed tomography (CT). Prompt referral and good teamwork are essential at every stage of management. Many advances in diagnosis and treatment have the potential to improve outcome in lung cancer. These demand a high degree of specialisation and multidisciplinary care. Such recommendations have appeared in guidelines published in the 1990s (SMAC, 1994; BTS, 1998; NHSE, 1998; SIGN, 1998). Positron emission tomography (PET) imaging became clinically available in a limited number of centres in the mid-1990s. It is a useful tool for refining the diagnosis and staging in patients with possible lung cancer. More recent guidelines recommend that patients who are staged as candidates for surgery or radical radiotherapy on CT should have an ^18^F-deoxyglucose-PET (FDG-PET) scan to look for involved intrathoracic lymph nodes and distant metastases (NICE, 2005). However, solitary extrapulmonary focal FDG accumulation may be due to a benign tumour or to inflammation. (Lardinois *et al*, 2005).

## Treatment of lung cancer between 1985 and 2000

Management of a patient with lung cancer depends largely on tumour type, extent of disease, general performance status of the patient and any significant comorbidity.

Surgery is the treatment of choice for operable patients with resectable Stage I and II NSCLC (Reif *et al*, 2000). Surgery also has an important role in managing selected patients with resectable Stage IIIA disease, sometimes in the context of combined modality therapy (Mountain, 1988; Reif *et al*, 2000). However, fewer than 20% of NSCLC patients have disease that is resectable at presentation (Martini and Flehinger, 1987). In spite of the intention to consider all patients with Stage I and II disease for surgery, there are those who, although technically operable, either decline surgery or are considered inoperable because of poor respiratory reserve, cardiovascular disease or general frailty. These ‘medically inoperable’ patients may be offered radical radiotherapy (RCR, 1999). Continuous hyperfractionated accelerated radiotherapy (CHART) compared with conventional radiotherapy gives a significant improvement in survival for patients with NSCLC (Saunders *et al*, 1997), although in 1998, CHART was not available in most UK centres.

However, in the majority of patients with NSCLC, advanced disease within the chest or metastatic disease precludes potentially curative treatment. For these patients, the aim of treatment is primarily palliative. In the 1980s and for most of the 1990s, there was substantial disagreement, both nationally and internationally, about the role of chemotherapy in addition to palliative radiotherapy and active supportive care (Carroll *et al*, 1986; Aisner and Belani, 1993; SMAC, 1994).

A meta-analysis of chemotherapy in NSCLC demonstrated a survival benefit in favour of cisplatin-based chemotherapy that reached conventional levels of significance when used with radical radiotherapy and with best supportive care (NSCLCCG, 1995). The most common treatment for NSCLC in the United Kingdom in the 1990s was cisplatin-based, usually mitomycin C, vinblastine and cisplatin (MVP) (Waters and O'Brien, 2002).

The mainstay of treatment in SCLC is combination chemotherapy. Commonly used combination chemotherapy regimes in the 1980s and 1990s include cyclophosphamide, doxorubicin and vincristine (CAV), cisplatin and etoposide (PE) and carboplatin and etoposide (CE). In limited SCLC, chemotherapy combined with thoracic radiotherapy yields 50–85% complete response rates, a median survival duration of 12–20 months and two year disease-free survival rates of 15–40% (Albain *et al*, 1990; Arriagada *et al*, 1992; Turrisi *et al*, 1999). Local treatments such as resection and radiotherapy have a limited effect in extensive SCLC (Souhami and Law, 1990) so the most widely accepted option for treating extensive disease is also with combination platinum-based chemotherapy. Despite diverse strategies for the treatment of patients with extensive SCLC, the results of phase III trials over the past 25 years have shown only a 2-month prolongation in median survival time between patients treated with different regimens (Chute *et al*, 1999). A more recent advance has been that prophylactic cranial irradiation significantly improves survival and disease-free survival for patients with SCLC in complete remission following chemotherapy (Auperin *et al*, 1999).

## Lung cancer survival trends in england and wales

The key trends in lung cancer survival as reported by Coleman *et al* (2004) are that 5-year survival for lung cancer increased marginally (on average 0.1% every 5 years) for men and women diagnosed in England and Wales over 1986–1999 but the average increase every 5 years during the 1990s was not statistically significant. Five-year survival for lung cancer patients diagnosed during 1996–1999 was 6% in men and women, not significantly better than for patients diagnosed a decade or so earlier. However, 1-year survival rates have risen from 15 to 25% for men and 13 to 26% for women diagnosed between 1971–1975 (Coleman *et al*, 1999) and 2000–2001 (Coleman *et al*, 2004).

There is good evidence that active treatment with surgery, radiotherapy or chemotherapy may improve survival in lung cancer and should not be denied to patients who might benefit (Brown *et al*, 1996; Fergusson *et al*, 1996; Muers and Haward, 1996). Higher rates and improving survival (5-year relative survival up to 14%) reported by some European countries must raise the possibility that opportunities exist for improvement. The EUROCARE-4 study identified considerable geographic variation of 12.3% in the 5-year survival rate for lung cancer across Europe, with the highest rates found in the Nordic region and central Europe, intermediate rates in southern Europe, lower rates in the United Kingdom and Ireland and the lowest rates in eastern Europe (Berrino *et al*, 2007).

Survival of NSCLC is largely dependent upon successful surgery. However, patients presenting with symptoms suggestive of lung cancer often experience delay at every stage of the referral process. The mean total delay experienced by patients from presentation to surgery was 109 days, including 1 month before initial referral to a specialist, and 2 months before subsequent referral to a surgeon (Billing and Wells, 1996). The annual national summary of resections for lung cancer carried out by all UK cardiothoracic surgeons shows a stable but lower figure of 10% (SCSGBI, 1994) compared with other European countries and the United States. There is therefore concern that many patients with operable tumours may be denied the chance of curative surgery.

There is an association between higher proportions of patients with a histological diagnosis and improved survival rates. An optimal proportion of patients with a histological diagnosis is generally considered to be around 80%. However, not all cases are confirmed histologically. Up until 1990, the rate was 50–60% (Connolly *et al*, 1990; Watkin *et al*, 1990), but by 1992–1994 this had improved to 70% in the Northern and Yorkshire region (NYCRIS, 1999). The rate of histological diagnosis decreases with age to 55% in patients aged 75–84 years (Kesson *et al*, 1998).

Despite evidence that chemotherapy may improve survival in some groups of lung cancer patients, only a small proportion of patients in the United Kingdom with NSCLC have been receiving chemotherapy (Clegg *et al*, 2001). For example, only 12% of people diagnosed with lung cancer in Wales in 1996 were given chemotherapy (WTS, 2000). There remained a widespread belief that chemotherapy for lung cancer was toxic and ineffective and a survey of clinicians who treated lung cancer in the United Kingdom found little support for chemotherapy (Crook *et al*, 1997).

Geographical differences exist in lung cancer survival. A study of over 24 500 cases of lung cancer between 1986 and 1994 found notable differences in 1- and 2-year survival between districts of residence in the Northern and Yorkshire region (NYCRIS, 1999). For example, 2-year survival in NSCLC varied by district between 7 and 18%. Five-year survival rates vary between different English health authorities, ranging from 2.2 to 8.9%, for patients diagnosed with lung cancer between 1993 and 1995 (DOH, 2002). There were wide variations in the rates of active treatment between districts and active treatment was strongly associated with improved survival (Cartman *et al*, 2002). A separate study of lung cancer patients diagnosed in southeast England between 1995 and 1999 found evidence for geographical inequality in the treatment given and patient survival (Jack *et al*, 2003). This study also found that patients whose first hospital attendance was at a radiotherapy centre survived longer and that the geographical inequalities may be explained by variations in access to oncology services.

This clinical background against which the study by Coleman *et al* was performed would suggest scope for improvement in the management of lung cancer.

## Socioeconomic inequalities in lung cancer survival in england and wales

Coleman *et al* (2004) found that survival among men was significantly lower for the poor than for the rich (deprivation gap −1.4%), a wider gap than for men diagnosed during 1986–1990, although the 5-yearly increase in the gap was not itself significant. The deprivation gap in survival for women diagnosed during 1996–1999 was small, and unchanged from a decade earlier.

The socioeconomic inequality in lung cancer survival among men may reflect a higher proportion of patients from the more deprived socioeconomic groups being more likely to present with comorbidity related to smoking, such as chronic obstructive pulmonary disease and ischaemic heart disease. In the case of NSCLC, patients from the most deprived groups may present with more advanced disease. Such factors may influence treatment, particularly surgery rates. Residents of a more deprived area may also be less likely to receive any active treatment, chemotherapy or radiotherapy (NYCRIS, 1999; Jack *et al*, 2003). Lower socioeconomic groups tend to use NHS services less in relation to need – this may reflect longer travel time, greater travel cost, lower car ownership, time constraints, differences in knowledge or beliefs about the need for medical attention (Dixon *et al*, 2003). However, these factors would also be expected to influence the deprivation gap in survival for women.

## Recent developments in lung cancer treatment

There have been many significant developments in the treatment of lung cancer since the turn of the century. The proportion of patients with NSCLC receiving active treatment has increased. There is strong evidence to recommend adjuvant chemotherapy for patients after resection of early NSCLC (Visbal *et al*, 2005). A further survival benefit has been identified in the treatment of Stage III NSCLC with chemoradiotherapy compared with radiotherapy alone (Rowell and O'Rourke, 2004). First-line treatment of advanced NSCLC with third-generation regimens incorporating gemcitabine, paclitaxel, vinorelbine or docetaxel with a platinum-based drug, have replaced second-generation agents like mitomycin C and vinblastine. Addition of anti-angiogenic agents to chemotherapy has been shown to be beneficial for patients with non-squamous NSCLC, although this is unlikely to be considered cost-effective within the NHS (Sandler *et al*, 2006). There is now a role for second-line treatment with docetaxel monotherapy (NICE, 2005). Epidermal growth factor receptor (EGFR) inhibition with erlotinib may prolong survival in patients with NSCLC after first-line or second-line chemotherapy (Shepherd *et al*, 2005). At the time of writing erlotinib is not generally available for NHS patients pending an imminent final decision by NICE. The Scottish Medicines Consortium accepted the case for erlotinib in the second-line treatment of NSCLC in May 2006 (SMC, 2006).

## Conclusion

Although cancer survival improved for most cancers in both sexes during the 1990s, the figures for lung cancer make for bleak reading. The need to standardise cancer care and treatment has been recognised by the Department of Health (DOH, 1995). This was followed by guidelines published in the 1990s aimed at improving outcomes in lung cancer (SMAC, 1994; BTS, 1998; NHSE, 1998; SIGN, 1998). However, it is unlikely that these initiatives will have significantly influenced the management of lung cancer between 1996 and 1999. There is usually a substantial time lag from publication of guidance to its implementation and any subsequent improvement in outcomes.

There have since been further initiatives like the NHS Cancer Plan (DOH, 2000). This was the first ever comprehensive strategy linking cancer prevention, diagnosis, treatment, care and research. The strategy was updated in 2004 in the NHS Cancer Plan and The New NHS. There have been many positive developments such as the emergence of the lung cancer specialist nurse service, the creation of lung cancer multidisciplinary teams, and the improvement in the evidence base for treatment, especially the third-generation chemotherapy agents. Developments in technology, such as FDG-PET scanning in disease staging, and the greater availability of CHART for the delivery of radical radiotherapy in suitable patients should be highlighted. These elements have been incorporated into detailed national guidelines for the diagnosis and treatment of lung cancer (NICE, 2005).

The study by Coleman *et al* provides baseline data to evaluate the efficacy of the restructuring of cancer services in England and Wales.
